# Severe SARS-CoV-2 infection as a marker of undiagnosed cancer: a population-based study

**DOI:** 10.1038/s41598-023-36013-7

**Published:** 2023-05-30

**Authors:** Adeline Dugerdil, Laura Semenzato, Alain Weill, Mahmoud Zureik, Antoine Flahault

**Affiliations:** 1 Institute of Global Health, Faculty of Medicine, University of Geneva, Campus Biotech, Chemin des Mines 9, 1202 Geneva, Switzerland; 2 EPI-PHARE Scientific Interest Group in Epidemiology of Health Products from the French National Agency for the Safety of Medicines and Health Products and the French National Health Insurance, 93285 Saint-Denis Cedex, France

**Keywords:** Oncology, Viral infection, Epidemiology

## Abstract

No study has yet investigated if a severe SARS-CoV-2 infection represents a marker of an undiagnosed cancer. This population-based study, using the SNDS database, identified from 02/15/2020 to 08/31/2021, 41,302 individuals hospitalized in intensive care unit due to SARS-CoV-2 (ICU-gr) and 713,670 control individuals not hospitalized for SARS-CoV-2 (C-gr). Individuals were matched according to year of birth, sex and French department. The cancer incidence was compared in the two groups during the follow-up period (index date-12/31/2021), using Cox proportional hazards models adjusted on matching variables, socioeconomic characteristics and comorbidities. In the ICU-gr, 2.2% (n = 897) was diagnosed with a cancer in the following months, compared to 1.5% (n = 10,944) in the C-gr. The ICU-gr had a 1.31 higher risk of being diagnosed with a cancer following hospital discharge compared to the C-gr (aHR 1.31, 95% CI 1.22–1.41). A global similar trend was found when competing risk of death was taken into account (aHR 1.25, 95% CI 1.16–1.34). A significant higher risk was found concerning renal (aHR 3.16, 95% CI 2.33–4.27), hematological (aHR 2.54, 95% CI 2.07–3.12), colon (aHR 1.72, 95% CI 1.34–2.21), and lung (aHR 1.70, 95% CI 1.39–2.08) cancers. This suggests that a severe SARS-CoV-2 infection may represent a marker of an undiagnosed cancer.

## Introduction

Since the first case of Severe Acute Respiratory Syndrome-Coronavirus 2 (SARS-CoV-2) in China in December 2019, the World Health Organization has recorded more than 580 million infections and more than 6.4 million deaths worldwide by the end of August 2022^1^.

As highlighted in a French study based on 66 million people, risk factors such as older age, male sex and numerous chronic conditions (including active cancers) are associated with a higher risk of hospitalization and death from SARS-CoV-2 infection^2^. Furthermore, vaccinated patients with active cancers have an increased risk of in-hospital death^3^. On an international level, numerous studies demonstrated that cancer patients are at higher risk of severe outcome when infected with SARS-CoV-2^4–11^. For example, an English study (OpenSAFELY database, including more than 17 million individuals) showed that hematological cancer patients were up to 2.5 times more likely to die from a SARS-CoV-2 infection^12^. Sinha and colleagues explain the greater fragility to SARS-CoV-2 in cancer patients as a result of six main elements: older age, increased expression of the ACE2 receptor, increased expression of the TMPRSS2 protease, underlying immunosuppression (due to cancer and/or anti-cancer treatments), significant inflammatory response and cancer-induced pro-coagulant state^13^.

The question one might ask is whether the severity of SARS-CoV-2 infection for individuals hospitalized in intensive care unit (ICU) is a marker or a result of underlying immunosuppression. Regarding the link between immunosuppression and new cancers, a meta-analysis (including 444,172 HIV/AIDS and 31,977 transplant patients), showed that these patients had an increased risk of developing cancers because of immunosuppression, particularly for cancers of infectious etiology (for example Hodgkin's lymphoma)^14^. Other studies support these results, either for stem cell transplant patients^15–17^ or HIV patients^18^. As explained by Al-Adra and colleagues, several pathophysiological mechanisms could explain the increased susceptibility of transplant patients to cancer: the loss of immune system control over oncogenic viruses, the accumulation of mutations no longer recognized by the immune system, and the direct effect of some immunosuppressive treatments^19^.

With the evidence that cancer is a risk factor for severe SARS-Cov-2 infection and being aware of the link between immunosuppression and the development of new cancers, the research question which motivated this study was the following: Is severe SARS-CoV-2 infection a marker of an undiagnosed cancer already present at the time of SARS-CoV-2 infection?

## Methodology

### Data sources

This study is based on the SNDS database (“*Système National des Données de Santé”*), a medico-administrative database which includes healthcare reimbursements data of the whole French population (67 million inhabitants) and which has been extensively used for pharmaco-epidemiology studies^2,3,20^. The SNDS database is subdivided into two distinct sub-databases, the DCIR database (“*Datamart de Consommation Inter Régimes”*) and the PMSI database (“*Programme de Médicalisation des Systèmes d'Information*”). Since 2006, the existence of a unique and anonymized identifier makes it possible to link the information contained in these two sub-databases. The DCIR contains information on the reimbursement of ambulatory medical care (including ambulatory medical care, laboratory tests and drugs according to the International Anatomical Therapeutic Chemical classification system). The PMSI contains information on the admission and discharge dates of any hospitalization in public or private hospital establishment in France, as well as the medical diagnoses related to the hospitalization (coded according to the ICD-10 classification, main medical or surgical procedures classified according to the “*Classification Commune des Actes Médicaux*”). Regarding the identification of individuals’ comorbidities, a specific tool was developed from the DCIR and the PMSI databases (“*Cartographie des Pathologies et des Dépenses*”), allowing the identification of pathologies from medical algorithms (based on the reasons for hospitalization, diagnoses of long-term illnesses and the reimbursement of specific treatments), for the previous 5 years^21^.

ICU hospitalized individuals were included from February 15, 2020 until August 31, 2021 (beginning of the SARS-CoV-2 pandemic, end of the 4th wave in France). Regarding the duration of follow-up, the index date was the admission date of hospitalization of ICU hospitalized individuals (same date for matched control individuals), with a follow-up end date on December 31, 2021, allowing a minimum follow-up of 4 months for individuals admitted to hospital in August 2021. All data was collected between March and June 2022 (reason why the follow-up end date was set for end of 2021).

### Inclusion and exclusion criteria

**Figure 1:**
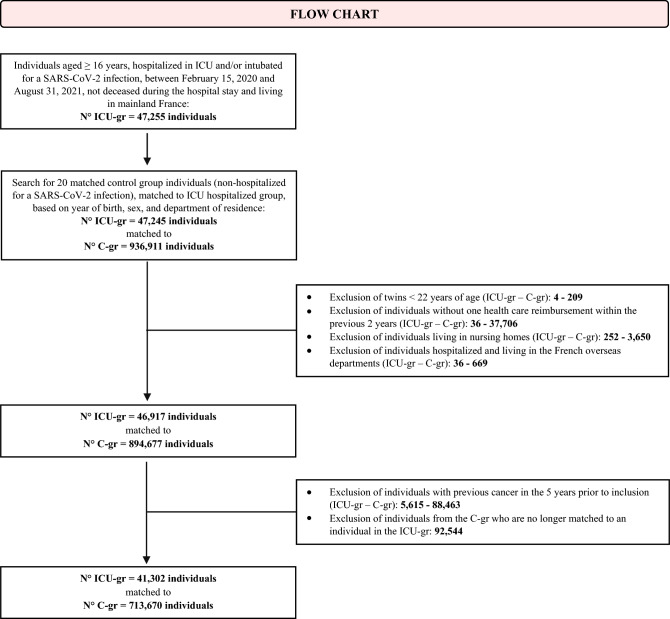
Flow Chart of the study. *ICU-gr* ICU hospitalized group, *C-gr* matched control group.

We collected data on individuals aged ≥ 16 years, living in mainland France, having benefited from at least one health care reimbursement in the 2 years preceding the index date, with no history of cancer in the previous 5 years. Individuals living in nursing homes and twins < 22 years were excluded (Fig. 1; Supplementary Table S1 online). Individuals were included in two groups, the ICU hospitalized group (ICU-gr) and the matched control group (C-gr). The two groups were matched on the basis of year of birth, sex, and French department (n = 95). Each individual from the ICU-gr was matched with between six (minimum) and 20 (maximum) individuals belonging to the C-gr (average number 17). We matched to a large number of controls (a maximum of 20 controls was deliberately chosen) in order to increase the representativeness of the C-gr when compared to the ICU-gr.

### Sociodemographic characteristics and co-variables

The following sociodemographic characteristics were taken into account: age, sex, and region of residence. The social deprivation index was used as a measure of the socio-economic status. The following co-variables were analyzed: various comorbidities, addictive disorders, vaccination status for SARS-CoV-2, and immunosuppressive/oral corticoid treatment (Table 1). The variables are defined in a previous article^2^, as the social deprivation index^22^.

**Table 1:** Sociodemographic characteristics and comorbidities of the ICU hospitalized group and the matched control group.

	Matched control group		ICU hospitalized group	
	Absolute number	Mean (standard deviation) or %	Absolute number	Mean (standard deviation) or %
Total	713,670	–	41,302	–
Sociodemographic characteristics				
Mean age in years (standard deviation)	–	60.0 (12.8)	–	60.8 (12.8)
Age category in years				
16–39	53,065	7.4%	2885	7.0%
40–49	85,666	12.0%	4641	11.2%
50–59	176,699	24.8%	9648	23.4%
60–69	220,258	30.9%	12,726	30.8%
70–79	151,745	21.3%	9620	23.3%
≥ 80	26,237	3.7%	1782	4.3%
Sex				
Female	236,964	33%	13,572	33%
Male	476,706	67%	27,730	67%
Region of residence				
Ile-de-France	202,880	28.4%	11,801	28.6%
Grand Est	68,431	9.6%	3936	9.5%
Hauts-de-France	72,895	10.2%	4175	10.1%
Auvergne-Rhône-Alpes	89,737	12.6%	5212	12.6%
Bourgogne-Franche-Comté	26,391	3.7%	1539	3.7%
Centre-Val de Loire	24,533	3.4%	1402	3.4%
Provence-Alpes-Côte d'Azur	73,288	10.3%	4289	10.4%
Occitanie	52,909	7.4%	3065	7.4%
Nouvelle-Aquitaine	34,467	4.8%	1984	4.8%
Normandie	27,295	3.8%	1562	3.8%
Pays de la Loire	23,948	3.4%	1366	3.3%
Bretagne	14,315	2.0%	816	2.0%
Corse	2581	0.4%	155	0.4%
Social deprivation index (quintiles)				
1: least deprived	168,864	23.7%	7829	19.0%
2	136,032	19.1%	7382	17.9%
3	129,750	18.2%	7801	18.9%
4	125,011	17.5%	7529	18.2%
5: most deprived	140,862	19.7%	10,118	24.5%
Missing data	13,151	1.8%	643	1.6%
Addictive disorders				
Smoking cessation program	35,493	5.0%	1565	3.8%
Alcohol related disorders	12,618	1.8%	617	1.5%
Opioid related disorders	2560	0.4%	119	0.3%
SARS-CoV-2 vaccination before index date				
Unvaccinated	617,392	86.5%	39,378	95.3%
1 dose	49,178	6.9%	1600	3.9%
2 doses	46,975	6.6%	306	0.7%
3 doses	125	0.02%	18	0.04%
Immunosuppressive/corticoid treatment				
Immunosuppressive medication	6636	0.9%	997	2.4%
Oral corticosteroids medication	5271	0.7%	1272	3.1%
Prior cardiometabolic comorbidities				
Diabetes	77,408	10.8%	10,199	24.7%
Morbid obesity	5037	0.7%	836	2.0%
Dyslipidemia and lipid-lowering treatments	146,644	20.5%	11,832	28.6%
Inherited metabolic diseases or amyloidosis	1574	0.2%	145	0.4%
Hypertension	238,909	33.5%	20,127	48.7%
Coronary heart disease	43,696	6.1%	5052	12.2%
Obliterative arteriopathy of the lower limbs	12,305	1.7%	1181	2.9%
Heart rate and conduction disorders	33,706	4.7%	7295	17.7%
Heart failure	9692	1.4%	3402	8.2%
Cardiac valve diseases	9279	1.3%	1474	3.6%
Stroke	14,158	2.0%	1752	4.2%
Other cardiovascular diseases	6029	0.8%	574	1.4%
Prior respiratory comorbidities				
Chronic respiratory diseases (excluding CF)	93,978	13.2%	18,626	45.1%
Cystic fibrosis	30	0.00%	17	0.04%
Pulmonary embolism	2318	0.3%	423	1.0%
Prior inflammatory and skin comorbidities				
Inflammatory bowel disease	3997	0.6%	289	0.7%
Rheumatoid arthritis and related diseases	4325	0.6%	605	1.5%
Ankylosing spondylitis and related diseases	3249	0.5%	632	1.5%
Other inflammatory diseases	2827	0.4%	551	1.3%
Psoriasis	6606	0.9%	524	1.3%
Prior psychiatric and neurodegenerative comorbidities				
Neurotic/mood disorders, use of antidepressants	60,785	8.5%	4860	11.8%
Psychotic disorders, use of neuroleptics	15,246	2.1%	1634	4.0%
Use of anxiolytics	56,571	7.9%	4547	11.0%
Use of hypnotics	23,725	3.3%	2057	5.0%
Psychiatric disorders since childhood	375	0.1%	37	0.1%
Epilepsy	3726	0.5%	396	1.0%
Multiple sclerosis	1520	0.2%	139	0.3%
Paraplegia	1284	0.2%	208	0.5%
Myopathy or myasthenia	636	0.1%	141	0.3%
Parkinson disease	4071	0.6%	266	0.6%
Dementia (including Alzheimer's disease)	3985	0.6%	325	0.8%
Mental disability	1469	0.2%	170	0.4%
Other psychiatric illnesses	3588	0.5%	314	0.8%
Other neurological diseases	2793	0.4%	317	0.8%
Other comorbidities				
HIV infection	3265	0.5%	283	0.7%
Liver diseases	7407	1.0%	2467	6.0%
Pancreatic diseases	2610	0.4%	405	1.0%
Chronic dialysis	770	0.1%	315	0.8%
Kidney transplantation	798	0.1%	409	1.0%
Cardiac transplantation	29	0.00%	16	0.04%
Liver transplantation	47	0.01%	11	0.03%
Lung transplantation	32	0.00%	19	0.05%
Haemophilia/severe haemostasis disorders	787	0.1%	96	0.2%
Down syndrome	227	0.0%	130	0.3%
Other long-term condition	10,668	1.5%	3036	7.4%

*CF* cystic fibrosis.

### Outcome and censoring criteria

The outcome was the incidence of cancers in the two groups during the follow-up period. A cancer case was defined as any hospitalization for cancer or any long-term cancer-like condition needing health care reimbursement (including in situ cancers). The censoring criteria which required the exclusion of the individual (or the end of the follow-up) after the initial inclusion were the death of the individual (ICU-gr and C-gr), the outcome occurrence (ICU-gr and C-gr), and the hospitalization due to a SARS-CoV-2 infection (C-gr; 5177 control individuals in the C-gr, i.e. < 1%, were censored because they were hospitalized for a SARS-CoV-2 infection; 694 of these control individuals were then re-included in the ICU-gr). The censoring criteria were applied at the individual level and censoring was done at the first event that occurred. The death was recorded through death certificates registered in the database, which therefore included deaths from any cause.

### Statistical analysis

The categorical variables are reported as frequencies with percentages and the continuous variables reported as means with standard deviations. To study the association between severe SARS-CoV-2 infection and overall cancer, as well as the association with specific cancer sites, we conducted Cox proportional hazards models that were systematically adjusted on matching variables and with further adjustment for all the co-variables previously described. In secondary analyses, we excluded in situ cancers, lung cancers or events occurring during the SARS-CoV-2 hospital stay. The follow-up was also divided into two sub-periods, distinguishing the first 3 months from the rest of the period, to assess the consistency of the associations over time (Table 3). Analyses by sex and age groups were performed (Table 4). Analysis taking into account death as competing event was conducted using Cox cause-specific hazard method (Supplementary Table S5 online). Missing data in the database, which concerned only the social deprivation index, were analyzed as a separate group (small number of missing data: 1.8% in C-gr, 1.6% in ICU-gr). All statistical analyses were performed using SAS software, version 9.4 (SAS Institute Inc.).

### Regulatory approval and ethical aspects

The French National Health Data System (SNDS) is a medico-administrative database containing all the healthcare reimbursements of the French population. EPI-PHARE has permanent regulatory access to the data from the SNDS via its constitutive bodies ANSM and CNAM. This permanent access is given in accordance with the French Decree No. 2016-1871 of December 26, 2016 relating to the processing of personal data called the "National Health Data System"^23^ and French law articles Art. R. 1461-13^24^ and 14^25^. All requests in the database were made by duly authorized people. In accordance with the permanent regulatory access granted to EPI-PHARE via ANSM and CNAM, this work did not require the approval from the French Data Protection Authority (CNIL) nor the approval from the ethics committee/institutional review board. The study was registered on the study register of EPI-PHARE concerning studies from SNDS data under the reference [EP-0376]. In accordance with data protection legislation and the French regulation, the authors cannot publicly release the data from the SNDS. However, any person or structure, public or private, for-profit or non-profit, is able to access SNDS data upon authorization from the French Data Protection Office (CNIL), in order to carry out a study, research or an evaluation in the public interest (https://www.snds.gouv.fr/SNDS/Processus-d-acces-aux-donnees and https://www.indsante.fr/).

## Results

Between February 15, 2020, and August 31, 2021, 41,302 individuals were hospitalized in the ICU in France due to a SARS-CoV-2 infection. These individuals were matched with 713,670 individuals who were not hospitalized for a SARS-CoV-2 infection (Fig. 1).

### Sociodemographic characteristics and comorbidities

Sociodemographic characteristics are detailed in Table 1. The mean age was 60.8 years in the ICU-gr (standard deviation (SD) 12.8), 60.0 years in the C-gr (SD 12.8), and 67% of individuals were men. More individuals from the ICU-gr were in the most deprived class (ICU-gr 24.5%; C-gr 19.7%). Smoking cessation program was slightly more prevalent in the C-gr (ICU-gr 3.8%; C-gr 5.0%). The majority of individuals of the two groups were not vaccinated against SARS-Cov-2 (without any vaccination dose received at the index date), even if unvaccinated individuals were more prevalent in the ICU-gr (ICU-gr 95.3%; C-gr 86.5%). Only 0.8% of individuals in the ICU-gr (n = 324) and 6.6% of individuals in the C-gr (n = 47,100) had received at least two doses of vaccine against SARS-CoV-2 at that time. However, SARS-CoV-2 vaccines were available to the French population only since December 27, 2020. More than twice as many individuals in the ICU-gr were under immunosuppressive therapy compared to the C-gr (ICU-gr 2.4%; C-gr 0.9%) and more individuals of the ICU-gr were under oral corticoid treatment (which includes any oral dose) compared to the C-gr (ICU-gr 3.1%; C-gr 0.7%). Individuals in the ICU-gr had more comorbidities overall compared to individuals in the C-gr. The median follow-up period was 327 days for the ICU-gr (interquartile range (IQR) 257–444 days) and 340 days for the C-gr (IQR 267–457 days).

### Cancer incidence in the two groups

In total, 897/41,302 individuals (2.2%) in the ICU-gr and 10,944/713,670 individuals (1.5%) in the C-gr were diagnosed with a cancer. The mean age at cancer diagnosis was 68.0 years (SD 9.3). The repartition of cancers according to cancer site is detailed in the Supplement (Supplementary Table S2 online). The median follow-up time for individuals to present the outcome was 168 days in the ICU-gr (IQR 73–270 days) and 200 days in the C-gr (IQR 99–322 days).

Using a Cox model adjusted only for age and sex, individuals in the ICU-gr had a 1.45 higher risk of being diagnosed with a cancer during the follow-up period compared to the C-gr (aHR 1.45, 95% CI 1.36–1.55). With a multivariable model (taking into account all the co-variables in Table 1), the adjusted HR was 1.31 for the ICU-gr (95% CI 1.22–1.41) (Table 2). The association obtained between the outcome and the exposure is relatively stable (same order of magnitude) between univariable and multivariable models, as is the range of the 95% confidence interval (Table 2). Similar results were observed when in situ cancers were excluded (aHR 1.32, 95% CI 1.23–1.42) or when lung cancers were excluded (aHR 1.27, 95% CI 1.18–1.37) (Supplementary Table S3 online).

**Table 2:** Occurrence of overall cancer in the ICU hospitalized group and the matched control group. The multivariable model (aHR) was adjusted for all variables presented in Table 1.

	Absolute number (%), without cancer	Absolute number (%), with cancer	Univariable model HR (95% CI)	Multivariable model aHR (95% CI)
Group				
Matched control group	702,726 (94.6%)	10,944 (92.4%)	1	1
ICU hospitalized group	40,405 (5.4%)	897 (7.6%)	1.49 (1.39–1.60)	1.31 (1.22–1.41)
Sociodemographic characteristics				
Age category in years				
16–39	55,872 (7.5%)	78 (0.7%)	1	1
40–49	89,977 (12.1%)	330 (2.8%)	2,52 (1.97–3.22)	2.33 (1.82–2.99)
50–59	184,777 (24.9%)	1570 (13.3%)	5.69 (4.53–7.14)	5.01 (3.99–6.29)
60–69	228,701 (30.8%)	4283 (36.2%)	12.22 (9.77–15.28)	10.28 (8.21–12.88)
70–79	156,793 (21.1%)	4572 (38.6%)	18.66 (14.91–23.34)	15.31 (12.22–19.18)
≥ 80	27,011 (3.6%)	1008 (8.5%)	23.04 (18.30–29.01)	18.97 (15.03–23.95)
Sex				
Female	247,468 (33.3%)	3068 (25.9%)	1	1
Male	495,663 (66.7%)	8773 (74.1%)	1.35 (1.30–1.41)	1.38 (1.32–1.44)
Regions of residence				
Île-de-France	211,833 (28.5%)	2848 (24.1%)	1	1
Grand Est	70,956 (9.5%)	1411 (11.9%)	1.39 (1.30–1.48)	1.15 (1.08–1.23)
Hauts-de-France	75,816 (10.2%)	1254 (10.6%)	1.32 (1.23–1.41)	1.17 (1.09–1.25)
Auvergne-Rhône-Alpes	93,322 (12.6%)	1627 (13.7%)	1.40 (1.31–1.48)	1.14 (1.07–1.21)
Bourgogne-Franche-Comté	27,395 (3.7%)	535 (4.5%)	1.49 (1.36–1.63)	1.13 (1.03–1.24)
Centre-Val de Loire	25,591 (3.4%)	344 (2.9%)	1.09 (0.98–1.22)	0.95 (0.85–1.06)
Provence-Alpes-Côte d'Azur	76,353 (10.3%)	1224 (10.3%)	1.40 (1.31–1.50)	1.17 (1.10–1.26)
Occitanie	55,140 (7.4%)	834 (7.0%)	1.30 (1.20–1.40)	1.08 (1.00–1.17)
Nouvelle-Aquitaine	35,843 (4.8%)	608 (5.1%)	1.49 (1.36–1.62)	1.25 (1.14–1.36)
Normandie	28,399 (3.8%)	458 (3.9%)	1.37 (1.24–1.52)	1.17 (1.06–1.29)
Pays de la Loire	24,915 (3.4%)	399 (3.4%)	1.36 (1.22–1.51)	1.21 (1.09–1.35)
Bretagne	14,886 (2.0%)	245 (2.1%)	1.38 (1.21–1.58)	1.18 (1.03–1.35)
Corse	2682 (0.4%)	54 (0.5%)	1.69 (1.29–2.22)	1.25 (0.96–1.64)
Social deprivation index (quintiles)				
1: least deprived	174,021 (23.4%)	2672 (22.6%)	1	1
2	141,168 (19.0%)	2246 (19.0%)	1.08 (1.02–1.14)	1.00 (0.94–1.06)
3	135,350 (18.2%)	2201 (18.6%)	1.11 (1.05–1.18)	0.98 (0.92–1.04)
4	130,405 (17.5%)	2135 (18.0%)	1.11 (1.05–1.18)	0.96 (0.91–1.02)
5: most deprived	148,630 (20.0%)	2350 (19.8%)	1.06 (1.01–1.12)	0.93 (0.88–0.99)
Unknown	13,557 (1.8%)	237 (2.0%)	1.19 (1.04–1.36)	1.00 (0.87–1.14)
Addictive disorders				
Smoking cessation program	36,285 (4.9%)	773 (6.5%)	1.42 (1.32–1.52)	1.34 (1.24–1.45)
Alcohol related disorders	12,873 (1.7%)	362 (3.1%)	1.80 (1.62–2.00)	1.63 (1.45–1.82)
Opioid related disorders	2651 (0.4%)	28 (0.2%)	0.67 (0.47–0.98)	0.95 (0.65–1.38)
SARS-CoV-2 vaccination before index date				
Unvaccinated	645,891 (86.9%)	10,879 (91.9%)	1	1
1 dose	50,213 (6.8%)	565 (4.8%)	1.16 (1.07–1.27)	1.00 (0.92–1.09)
2 doses	46,884 (6.3%)	397 (3.4%)	1.15 (1.04–1.27)	1.04 (0.94–1.15)
3 doses	143 (0.0%)	–	–	–
Immunosuppressive/corticoid treatment				
Immunosuppressive medication	7474 (1.0%)	159 (1.3%)	1.37 (1.17–1.60)	1.14 (0.93–1.39)
Oral corticosteroids medication	6360 (0.9%)	183 (1.5%)	1.80 (1.56–2.08)	1.20 (1.02–1.42)
Prior cardiometabolic comorbidities				
Diabetes	85,644 (11.5%)	1963 (16.6%)	1.50 (1.43–1.57)	0.94 (0.89–0.99)
Morbid obesity	5803 (0.8%)	70 (0.6%)	0.78 (0.61–0.98)	0.85 (0.67–1.07)
Dyslipidemia and lipid-lowering treatments	154,623 (20.8%)	3853 (32.5%)	1.78 (1.71–1.85)	1.02 (0.97–1.07)
Inherited metabolic diseases or amyloidosis	1687 (0.2%)	32 (0.3%)	1.18 (0.83–1.67)	0.87 (0.61–1.22)
Hypertension	252,859 (34.0%)	6177 (52.2%)	2.05 (1.98–2.12)	1.20 (1.15–1.25)
Coronary heart disease	47,443 (6.4%)	1305 (11.0%)	1.77 (1.67–1.87)	0.96 (0.90–1.03)
Obliterative arteriopathy of the lower limbs	12,945 (1.7%)	541 (4.6%)	2.66 (2.44–2.90)	1.48 (1.36–1.62)
Heart rate and conduction disorders	39,788 (5.4%)	1213 (10.2%)	1.98 (1.87–2.10)	1.07 (1.00–1.14)
Heart failure	12,696 (1.7%)	398 (3.4%)	2.01 (1.82–2.22)	1.00 (0.90–1.11)
Cardiac valve diseases	10,449 (1.4%)	304 (2.6%)	1.83 (1.63–2.05)	0.99 (0.88–1.12)
Stroke	15,505 (2.1%)	405 (3.4%)	1.63 (1.48–1.81)	1.01 (0.91–1.11)
Other cardiovascular diseases	6404 (0.9%)	199 (1.7%)	1.94 (1.69–2.23)	1.16 (1.01–1.34)
Prior respiratory comorbidities				
Chronic respiratory diseases (excluding CF)	109,762 (14.8%)	2842 (24.0%)	1.71 (1.62–1.81)	1.20 (1.13–1.27)
Cystic fibrosis	47 (0.0%)	–	–	–
Pulmonary embolism	2668 (0.4%)	73 (0.6%)	1.71 (1.36–2.15)	1.14 (0.90–1.43)
Prior inflammatory and skin comorbidities				
Inflammatory bowel disease	4226 (0.6%)	60 (0.5%)	0.90 (0.70–1.16)	0.85 (0.66–1.10)
Rheumatoid arthritis and related diseases	4831 (0.7%)	99 (0.8%)	1.30 (1.07–1.59)	0.91 (0.73–1.13)
Ankylosing spondylitis and related diseases	3819 (0.5%)	62 (0.5%)	1.04 (0.81–1.33)	0.87 (0.67–1.12)
Other inflammatory diseases	3308 (0.4%)	70 (0.6%)	1.35 (1.07–1.71)	0.94 (0.74–1.20)
Psoriasis	6970 (0.9%)	160 (1.4%)	1.44 (1.23–1.68)	1.16 (0.99–1.35)
Prior psychiatric and neurodegenerative comorbidities				
Neurotic and mood disorders, use of antidepressants	64,548 (8.7%)	1097 (9.3%)	1.10 (1.03–1.17)	0.91 (0.85–0.98)
Psychotic disorders, use of neuroleptics	16,625 (2.2%)	255 (2.2%)	0.99 (0.87–1.12)	0.99 (0.87–1.13)
Use of anxiolytics	59,932 (8.1%)	1186 (10.0%)	1.29 (1.22–1.37)	1.03 (0.96–1.10)
Use of hypnotics	25,198 (3.4%)	584 (4.9%)	1.50 (1.38–1.63)	1.11 (1.02–1.21)
Psychiatric disorders since childhood	411 (0.1%)	1 (0.0%)	0.16 (0.02–1.14)	0.25 (0.04–1.79)
Epilepsy	4057 (0.5%)	65 (0.5%)	1.01 (0.79–1.29)	0.86 (0.67–1.10)
Multiple sclerosis	1645 (0.2%)	14 (0.1%)	0.56 (0.33–0.94)	0.62 (0.37–1.06)
Paraplegia	1459 (0.2%)	33 (0.3%)	1.46 (1.04–2.06)	1.31 (0.93–1.86)
Myopathy or myasthenia	756 (0.1%)	21 (0.2%)	1.77 (1.15–2.72)	1.41 (0.92–2.17)
Parkinson disease	4232 (0.6%)	105 (0.9%)	1.56 (1.29–1.89)	0.97 (0.79–1.17)
Dementia (including Alzheimer's disease)	4201 (0.6%)	109 (0.9%)	1.65 (1.36–1.99)	0.88 (0.72–1.08)
Mental disability	1621 (0.2%)	18 (0.2%)	0.72 (0.46–1.15)	0.93 (0.59–1.49)
Other psychiatric illnesses	3813 (0.5%)	89 (0.8%)	1.45 (1.18–1.79)	1.12 (0.89–1.40)
Other neurological diseases	3040 (0.4%)	70 (0.6%)	1.47 (1.16–1.86)	1.21 (0.96–1.54)
Other comorbidities				
HIV infection	3470 (0.5%)	78 (0.7%)	1.38 (1.10–1.72)	1.53 (1.22–1.91)
Liver diseases	9569 (1.3%)	305 (2.6%)	2.02 (1.80–2.26)	1.47 (1.31–1.66)
Pancreatic diseases	2935 (0.4%)	80 (0.7%)	1.71 (1.37–2.13)	1.23 (0.98–1.53)
Chronic dialysis	1042 (0.1%)	43 (0.4%)	2.66 (1.97–3.58)	1.57 (1.16–2.12)
Kidney transplantation	1173 (0.2%)	34 (0.3%)	1.90 (1.35–2.65)	1.18 (0.80–1.75)
Cardiac transplantation	44 (0.01%)	1 (0.01%)	1.37 (0.19–9.72)	1.02 (0.14–7.38)
Liver transplantation	56 (0.01%)	2 (0.02%)	2.31 (0.58–9.20)	0.99 (0.24–4.00)
Lung transplantation	50 (0.01%)	1 (0.01%)	1.33 (0.19–9.41)	0.77 (0.11–5.51)
Haemophilia/severe haemostasis disorders	859 (0.1%)	24 (0.2%)	1.80 (1.20–2.68)	1.46 (0.98–2.18)
Down syndrome	355 (0.05%)	2 (0.02%)	0.38 (0.09–1.50)	0.72 (0.18–2.89)
Other long-term condition	13,380 (1.8%)	324 (2.7%)	1.50 (1.35–1.68)	1.18 (1.05–1.32)

*CF* cystic fibrosis.

### Stratification according to the follow-up period

The association between the risk of being diagnosed with a cancer and exposure (ICU-gr vs C-gr) was stronger in the first 3 months of follow-up, starting at the index date (aHR 1.65, 95% CI 1.45–1.88), compared to the rest of the follow-up period (aHR 1.21, 95% CI 1.11–1.33). This result was confirmed even when lung cancers were excluded from the multivariable analysis (Period 1: aHR 1.59, 95% CI 1.38–1.83; Period 2: aHR 1.18, 95% CI 1.07–1.30) (Table 3).

**Table 3:** Stratification according to the follow-up period for overall cancers and without lung cancers. The multivariable model (aHR) was adjusted for all variables presented in Table 1.

	Global follow-up period			Period 1: first 3 months after hospital admission		Period 2: rest of the follow-up period	
	Total number	Cancer absolute number	MV model aHR (95% CI)	Cancer absolute number	MV model aHR (95% CI)	Cancer absolute number	MV model aHR (95% CI)
Global							
Matched control group/Total	713,670/754,972	10,944/11,841	1	2537/2810	1	6674/7197	1
ICU hospitalized group/Total	41,302/754,972	897/11,841	1.31 (1.22–1.41)	273/2810	1.65 (1.45–1.88)	523/7197	1.21 (1.11–1.33)
Without lung cancers							
Matched control group/Total	712,572/753,751	9944/10,723	1	2318/2556	1	6111/6571	1
ICU hospitalized group/Total	41,179/753,751	779/10,723	1.27 (1.18 – 1.37)	238/2556	1.59 (1.38 – 1.83)	460/6571	1.18 (1.07 – 1.30)

*MV model* multivariable model.

### Stratification according to age and sex

The association between exposure and the risk of cancer was stronger in women compared to men (aHR 1.69, 95% CI 1.48–1.93, and aHR 1.20, 95% CI 1.10–1.30, respectively) and among individuals younger than 60 years old compared to older individuals (aHR 1.78, 95% CI 1.52–2.09, and aHR 1.22, 95% CI 1.12–1.32, respectively). The strongest association was found in women under 60 years old (aHR 2.15, 95% CI 1.65–2.80) (Table 4).

**Table 4:** Estimated risk of cancer diagnosis stratified by age (all, < 60 years, ≥ 60 years) and sex (all, male, female). The multivariable model (aHR) was adjusted for all variables presented in Table 1.

	All aHR (95% CI)	Age < 60 years aHR (95% CI)	Age ≥ 60 years aHR (95% CI)
All (male and female)	1.31 (1.22–1.41)	1.78 (1.52–2.09)	1.22 (1.12–1.32)
Male	1.20 (1.10–1.30)	1.65 (1.35–2.02)	1.12 (1.02–1.23)
Female	1.69 (1.48–1.93)	2.15 (1.65–2.80)	1.57 (1.34–1.82)

We performed Cox multivariable models adjusted for all the variables cited in Table 1 in subpopulation as sensitivity analyses. For example, among women aged less than 60 years old, the ICU hospitalized group had a 2.15 higher risk of being diagnosed with a cancer compared to the matched control group (aHR 2.15, 95% CI 1.65–2.80).

### Occurrence of cancer according to cancer site

An analysis of cancer distribution according to cancer site is detailed in Table 5. The risk of being diagnosed with a cancer was significantly higher in the ICU-gr than in the C-gr regarding the following categories: renal cancer (aHR 3.16, 95% CI 2.33–4.27), hematological cancer (aHR 2.54, 95% CI 2.07–3.12), colon cancer (aHR 1.72, 95% CI 1.34–2.21), lung cancer (aHR 1.70, 95% CI 1.39–2.08), and other malignancies (aHR 1.18, 95% CI 1.04–1.35). Among the hematological cancer, ICU-gr had a significantly higher risk of being diagnosed with a leukemia (aHR 3.28, 95% CI 2.41–4.46), a myeloma (aHR 2.21, 95% CI 1.36–3.59), or a non-Hodgkin’s lymphoma (aHR 2.15, 95% CI 1.53–3.04), compared to the C-gr. No difference could be found between the two groups for the following cancers: Hodgkin’s lymphoma, melanoma, breast, prostate, rectal, liver, bladder, and uterine cancers (Table 5). Regarding the “other malignancies” category, more details can be found in the Supplement (Supplementary Table S2 online).

**Table 5:** Occurrence of cancer according to cancer site. The multivariable model (aHR) was adjusted for all variables presented in Table 1.

	Cancer diagnosis after hospital admission			
	Cancer in absolute number	Median follow-up in days (q1–q3)	Crude incidence/100,000 Pyr	Multivariable model aHR (95% CI)
Any malignancy				
Matched control group	10,944	200 (99–322)	1469	1
ICU hospitalized group	897	168 (73–270)	2192	1.31 (1.22–1.41)
Hematological cancer				
Matched control group	759	202 (106–327)	102	1
ICU hospitalized group	124	107 (50–233)	303	2.54 (2.07–3.12)
Non-Hodgkin's lymphoma				
Matched control group	300	210 (111–334)	40	1
ICU hospitalized group	43	111 (55–262)	105	2.15 (1.53–3.04)
Hodgkin's lymphoma				
Matched control group	32	194 (122–290)	4	1
ICU hospitalized group	2	204 (135–273)	5	1.03 (0.24–4.51)
Myeloma				
Matched control group	142	198 (92–305)	19	1
ICU hospitalized group	22	53 (6–118)	54	2.21 (1.36–3.59)
Leukemia				
Matched control group	285	201 (103–323)	38	1
ICU hospitalized group	57	137 (59–203)	139	3.28 (2.41–4.46)
Female breast cancer				
Matched control group	863	191 (98–320)	116	1
ICU hospitalized group	55	154 (92–218)	134	1.13 (0.85–1.50)
Prostate cancer				
Matched control group	2187	200 (97–323)	293	1
ICU hospitalized group	104	216 (117–299)	254	0.87 (0.71–1.06)
Colon cancer				
Matched control group	668	195 (103–335)	90	1
ICU hospitalized group	75	140 (42–271)	183	1.72 (1.34–2.21)
Rectal cancer				
Matched control group	247	219 (117–359)	33	1
ICU hospitalized group	12	175 (53–418)	29	0.91 (0.50–1.64)
Lung cancer				
Matched control group	1000	197 (105–313)	134	1
ICU hospitalized group	118	143 (74–244)	288	1.70 (1.39–2.08)
Liver cancer				
Matched control group	303	182 (88–298)	41	1
ICU hospitalized group	27	189 (82–310)	66	0.83 (0.55–1.26)
Bladder cancer				
Matched control group	581	202 (94–322)	78	1
ICU hospitalized group	42	143 (64–238)	103	1.09 (0.79–1.51)
Renal cancer				
Matched control group	302	204 (106–338)	41	1
ICU hospitalized group	58	140 (80–245)	142	3.16 (2.33–4.27)
Uterine cancer				
Matched control group	103	180 (109–299)	14	1
ICU hospitalized group	6	111 (20–268)	15	1.01 (0.43–2.39)
Malignant melanoma				
Matched control group	326	199 (108–302)	44	1
ICU hospitalized group	11	213 (80–302)	27	0.73 (0.40–1.34)
Other malignancy				
Matched control group	3581	201 (96–321)	481	1
ICU hospitalized group	265	202 (96–300)	648	1.18 (1.04–1.35)

The same analysis was performed, but with the follow-up starting only after hospital discharge. The ICU-gr had then a 1.17 higher risk of being diagnosed with a cancer compared to the C-gr (aHR 1.17, 95% CI 1.08–1.26). The results showed a similar trend for each category of cancer, apart for myeloma (aHR 1.21, 95% CI 0.67–2.21) (Supplementary Table S4 online). A final analysis was performed taking into account the competing risk of death with the multivariable model. The ICU-gr had then a 1.25 higher risk of being diagnosed with a cancer compared to the C-gr (aHR 1.25, 95% CI 1.16–1.34). The overall categories showed a similar trend, apart from other malignancies category (aHR 1.13, 95% CI 0.99–1.29) (Supplementary Table S5 online).

## Discussion

This large population-based study included 41,302 individuals hospitalized in ICU due to SARS-CoV-2 infection (between February 15, 2020 and August 31, 2021) and 713,670 control individuals. Among these individuals, 2.2% of the ICU-gr was diagnosed with a cancer compared to 1.5% in the C-gr. Individuals in the ICU-gr had a 1.31 higher risk of being diagnosed with a cancer compared to the C-gr. The association was stronger by limiting the follow-up period to the first 3 months, and among women. The ICU-gr had a significant higher risk of being diagnosed with a renal, hematological, colon, or a lung cancer, compared to the C-gr. No significant differences were found for the other sites of cancers.

To the best of our knowledge, to date no studies have been conducted on this issue. However, studies with similar design aiming to assess the risk of cancer following other diseases, such as herpes zoster, have already been conducted. For example, a study conducted in the United Kingdom, using the General Practice Research Database (including 74,029 individuals), demonstrated the link between individuals having had herpes zoster and the risk of them being diagnosed with cancer in the following years^26^.

This study cannot conclude on a causal effect of a severe SARS-COV-2 infection on the risk of developing a cancer in the following months. Cancer screening and diagnosis may indeed have been different between the two groups, leading to a detection bias. Individuals hospitalized in the ICU-gr may have benefited from more lung scans, used as a screening tool for lung cancers, and from more repetitive blood tests that allowed screening of hematological diseases. On the other hand, screening by PSA or mammography may have been less frequent during the ICU stay or at discharge, as this was not necessarily a priority for these patients. For the control group, individuals were probably able to benefit from a better screening for certain cancers as they did not experience serious health events and were in better health condition to receive these screenings. However, since individuals hospitalized in ICU for a SARS-CoV-2 infection had a 31% higher risk of being diagnosed with a cancer in an average of 168 days following the index date, a severe SARS-CoV-2 infection may represent a marker of an underlying undiagnosed cancer, especially as the association with the risk of being diagnosed with a cancer was stronger in the first 3 months following hospitalization. Therefore, a more systematic screening could be more efficient during this period of time. It should also be noted that identical multivariate analyses were performed taking into account follow-up starting only from hospital discharge. These additional results showed a 17% increased risk of being diagnosed with a cancer in the ICU-gr compared to the C-gr, which underlines the fact that even when the follow-up does not include the hospitalization period, a similar trend is confirmed despite the possible detection bias previously described. Furthermore, multivariate analyses were performed taking into account the competing risk of death, highlighting a global similar trend with a 25% increased risk of being diagnosed with a cancer in the ICU-gr compared to the C-gr.

Regarding cancer sites, renal, hematological, colon and lung cancers were most likely to be diagnosed following a severe SARS-CoV-2 infection. While it may be more intuitive to understand why some type of hematological cancer might impact the immune system, it may be more difficult to understand the link between renal or colon cancer and higher frailty to SARS-Cov-2 infection. Nevertheless, some recent studies have already confirmed the immune dysfunction associated with renal and colon cancers^27–29^, as well as the fact that any type of cancer may promote immune dysfunction^30^. This could represent one explanation to our findings.

### Strengths of the study

The main strength of this study is that the SNDS is a claims database that allowed us to analyze the risk of being diagnosed with a cancer from the comprehensive population without cancer history, thus limiting selection bias. Furthermore, a large number of individuals were included in the study, as the database includes the whole French population. In addition, all analyses were adjusted with a multivariable model to minimize confounding factors.

### Limitations of the study

This study had several limitations. Firstly, the definition of a severe SARS-CoV-2 infection was limited to individuals hospitalized in ICU. However, this allowed us to focus on the most severe cases of SARS-CoV-2 infections. Secondly, information was potentially wrongly classified for certain variables (obesity, tobacco dependence, alcohol related disorders), which are significantly underestimated in this database. For instance, it is possible that some patients who smoke were misclassified as non-smokers in the database, thus underestimating this variable. However, this should not substantially modify the association between the risk of being diagnosed with a cancer and the group of exposure, except probably for obesity. Thirdly, we did not have information on the medication of residents in nursing homes, which have their own pharmacy, and therefore did not identify their comorbidities exhaustively. For this reason and knowing that many of these patients were not admitted to hospital during the first wave of SARS-CoV-2 pandemic because of the hospital restrictions in place at this time in France, we excluded this subpopulation. Finally, our study may also have been affected by residual confounding factors due to differences between the two groups, although matching and adjustment for a high range of comorbidities have been done.

## Conclusion

In conclusion, this study is the first to suggest an association between severe SARS-CoV-2 infection and cancer diagnosis in the following months, suggesting that a severe SARS-CoV-2 infection may represent a marker of undiagnosed cancer. More research is needed to determine the nature of the relationship between an underlying cancer and a severe SARS-CoV-2 infection. Based on this future research, it would be necessary to discuss whether more targeted screening should be offered or not to this population of individuals.

## Supplementary Information

Supplementary Tables.

## Data Availability

The data that support the findings of this study are available from the French Data Protection Office (CNIL Commission Nationale de l’Informatique et des Libertes) via the French Health Data Hub (https://www.snds.gouv.fr/SNDS/Processus-d-acces-aux-donnees and https://www.health-data-hub.fr/) but restrictions apply to the availability of these data, which were used under license for the current study, and so are not publicly available. Data are however available from the authors upon reasonable request and with permission of the French Data Protection Office (CNIL Commission Nationale de l’Informatique et des Libertes). All methods were carried out in accordance with relevant guidelines and regulations. All experimental protocols were approved by a named institutional and/or licensing committee, as detailed in the paragraph below: EPI-PHARE has permanent regulatory access to the data from the French National Health Data System (SNDS) via its constitutive bodies ANSM and CNAM, in application of the provisions of the French Decree No. 2016-1871 of December 26, 2016 relating to the processing of personal data called the "National Health Data System", the French law articles Art. R. 1461-13 and R. 1461-14 from the French Public Health Code and the French Data Protection Authority (CNIL) decision CNIL-2016-316. All requests in the database were made by duly authorized people. In accordance with the permanent regulatory access granted to EPI-PHARE via ANSM and CNAM, this work did not require any specific approval from the CNIL. The study was registered on the study register of EPI-PHARE concerning studies from SNDS data under the reference [EP-0376]. The research group has permanent regulatory access to the data from the French National Health Data System (French decree No. 2016-1871 of December 26, 2016, on the processing of personal data called National Health Data System and French law articles Art. R. 1461-13 and 14) upon authorization from the French Data Protection Office (CNIL Commission Nationale de l’Informatique et des Libertés). No informed consent was required because the data are anonymized.
