# Supplementary material for: Severe SARS-CoV-2 infection as a marker of undiagnosed cancer: a population-based study
Source: Sci Rep. 2023 May 30;13:8729. doi: 10.1038/s41598-023-36013-7 (PMC10227779; doi:10.1038/s41598-023-36013-7)
Supplement: Supplementary file 1 — Supplementary Tables. [file 41598_2023_36013_MOESM1_ESM.pdf]

Supplementary tables for the article entitled "Severe SARS-CoV-2 infection as a marker of undiagnosed cancer – A population-based study"

Adeline Dugerdil[1§], MD; Laura Semenzato[2§], MSc; Alain Weill[2], MD; Mahmoud Zureik[2#], MD, PhD; and Antoine Flahault[1#], MD, PhD

[§]The work was distributed equally between the two first co-authors.

[#]The work was distributed equally between the two last co-authors.

[1]Institute of Global Health, Faculty of Medicine, University of Geneva, Geneva, Switzerland.

[2]EPI-PHARE Scientific Interest Group in Epidemiology of Health Products from the French National Agency for the Safety of Medicines and Health Products and the French National Health Insurance, 93285, Saint-Denis Cedex, France.

Supplementary tables:

1. Supplementary Table S1: Inclusion and exclusion criteria for the ICU hospitalized group and the matched control group.
2. Supplementary Table S2: Cancer repartition in the ICU hospitalized and the matched control group.
3. Supplementary Table S3: Occurrence of cancer in the ICU hospitalized group and the matched control group, without in situ cancers and without lung cancers.
4. Supplementary Table S4: Stratification according to cancer site, taking into account diagnoses made after hospital discharge.
5. Supplementary Table S5: Stratification according to cancer site, taking into account death as a competing risk.
6. Supplementary Table S6: List of codes used to classify cancers in the SNDS database.

Supplementary Table S1: Inclusion and exclusion criteria for the ICU hospitalized group and the matched control group.

	Inclusion criteria	Exclusion criteria
ICU hospitalized group (ICU-gr)	• Age ≥ 16 years<br>• Having been hospitalized and living in mainland France<br>• Having benefited from at least one health care reimbursement in the 2 years preceding the date of hospitalization<br>• Having been hospitalized in ICU and/or intubated, due to a SARS-CoV-2 infection, and not deceased during the hospital stay, between 15/02/2020 and 31/08/2021<br>• Having no history of cancer in the previous 5 years	• Age < 16 years<br>• Twin individuals < 22 years<br>• Missing value for sex or age<br>• Living in the French overseas departments<br>• Living in a nursing home<br>• Not having benefited from at least one health care reimbursement in the 2 years preceding the date of hospitalization<br>• Having been diagnosed with cancer at the time of hospital admission (ICU-gr) or at matching time (C-gr) / Having a history of cancer in the previous 5 years<br>• Deceased during hospitalization (ICU-gr) or during follow-up (C-gr)
Matched control group (C-gr)	• Age ≥ 16 years<br>• Living in mainland France<br>• Having benefited from at least one health care reimbursement in the 2 years preceding the date of hospitalization<br>• Not having been hospitalized for SARS-CoV-2 infection at the index date of the case (but may or may not have had SARS-CoV-2)<br>• Having no history of cancer in the previous 5 years<br>• Matching criteria to ICU-gr: year of birth, sex, French department (95 geographic area)	

Supplementary Table S2: Cancer repartition in the ICU hospitalized and the matched control group.

	Matched control group			ICU hospitalized group		
	Absolute number	% on cancer population	% on total population	Absolute number	% on cancer population	% on total population
**Cancer population/Total population included in the study**	**10,944/713,670**	..	**1.53%**	**897/41,302**	..	**2.17%**
**Median follow-up in days (q1 - q3), for individuals with cancer**	**200 (99 - 322)**	..	..	**168 (73 - 270)**	..	..
**Cancer site**						
Bladder cancer	581	5.3%	0.081%	42	4.7%	0.102%
Hematological cancer	759	6.9%	0.106%	124	13.8%	0.300%
N-H lymphoma	300	2.7%	0.042%	43	4.8%	0.104%
Hodgkin's lymphoma	32	0.3%	0.004%	2	0.2%	0.005%
Myeloma	142	1.3%	0.020%	22	2.5%	0.053%
Leukemia	285	2.6%	0.040%	57	6.4%	0.138%
Female breast cancer	887	8.1%	0.124%	55	6.1%	0.133%
Colon cancer	668	6.1%	0.094%	75	8.4%	0.182%
Liver cancer	303	2.8%	0.042%	27	3.0%	0.065%
Lung cancer	1,000	9.1%	0.140%	118	13.2%	0.286%
Melanoma	326	3.0%	0.046%	11	1.2%	0.027%
Prostate cancer	2,187	20.0%	0.306%	104	11.6%	0.252%
Rectal cancer	247	2.3%	0.035%	12	1.3%	0.029%
Renal cancer	302	2.8%	0.042%	58	6.5%	0.140%
Uterine cancer	103	0.9%	0.014%	6	0.7%	0.015%
Other malignancies	3,581	32.7%	0.502%	265	29.5%	0.642%
Digestive cancer	744	6.8%	0.104%	45	5.0%	0.109%
FGT cancer	115	1.1%	0.016%	11	1.2%	0.027%
MGT cancer	38	0.3%	0.005%	3	0.3%	0.007%
In situ cancer	302	2.8%	0.042%	14	1.6%	0.034%
Cancer of UE	319	2.9%	0.045%	27	3.0%	0.065%
ENT cancer	295	2.7%	0.041%	10	1.1%	0.024%
Bone cancer	16	0.1%	0.002%	2	0.2%	0.005%
Skin cancer	1,152	10.5%	0.161%	66	7.4%	0.160%
RT cancer	171	1.6%	0.024%	33	3.7%	0.080%
CNS cancer	161	1.5%	0.023%	15	1.7%	0.036%
Thyroid cancer	122	1.1%	0.017%	21	2.3%	0.051%
Soft tissue cancer	105	1.0%	0.015%	12	1.3%	0.029%
Urinary tract cancer	41	0.4%	0.006%	6	0.7%	0.015%

Abbreviations: NH = Non-Hodgkin, FGT = Female Genital Tract, MGT = Male Genital Tract, UE = Undetermined Etiology, ENT= Ear, Nose and Throat, RT = Respiratory Tract, CNS = Central Nervous System.

Supplementary Table S3: Occurrence of cancer in the ICU hospitalized group and the matched control group, without in situ cancers and without lung cancers. The multivariable model (aHR) was adjusted for all variables presented in Table 1.

	Multivariable model, adjusted hazard ratio (95% CI)	Multivariable model without in situ cancers, adjusted hazard ratio (95% CI)	Multivariable model without lung cancers, adjusted hazard ratio (95% CI)
**Group**			
Matched control group	1	1	1
ICU hospitalized group	1.31 (1.22 – 1.41)	1.32 (1.23 – 1.42)	1.27 (1.18 – 1.37)
**Sociodemographic characteristics**			
**Age category in years**			
16 - 39	1	1	1
40 - 49	2.33 (1.82 – 2.99)	2.47 (1.90 - 3.21)	2.23 (1.74 - 2.87)
50 - 59	5.01 (3.99 – 6.29)	5.59 (4.38 - 7.13)	4.65 (3.70 - 5.85)
60 - 69	10.28 (8.21 – 12.88)	11.53 (9.07 - 14.66)	9.52 (7.59 - 11.93)
70 - 79	15.31 (12.22 – 19.18)	17.15 (13.48 - 21.82)	14.31 (11.40 - 17.96)
≥ 80	18.97 (15.03 – 23.95)	21.40 (16.69 - 27.42)	18.02 (14.24 - 22.79)
**Sex**			
Female	1	1	1
Male	1.38 (1.32 – 1.44)	1.40 (1.34 - 1.46)	1.36 (1.30 - 1.42)
**Regions of residence**			
Île-de-France	1	1	1
Grand Est	1.15 (1.08 – 1.23)	1.13 (1.06 - 1.21)	1.17 (1.09 - 1.26)
Hauts-de-France	1.17 (1.09 – 1.25)	1.15 (1.07 - 1.23)	1.20 (1.11 - 1.29)
Auvergne-Rhône-Alpes	1.14 (1.07 – 1.21)	1.12 (1.05 - 1.19)	1.16 (1.08 - 1.24)
Bourgogne-Franche-Comté	1.13 (1.03 – 1.24)	1.12 (1.02 - 1.23)	1.14 (1.03 - 1.26)
Centre-Val de Loire	0.95 (0.85 – 1.06)	0.94 (0.84 - 1.05)	0.96 (0.85 - 1.08)
Provence-Alpes-Côte d'Azur	1.17 (1.10 – 1.26)	1.14 (1.06 - 1.22)	1.19 (1.10 - 1.28)
Occitanie	1.08 (1.00 – 1.17)	1.05 (0.96 - 1.13)	1.10 (1.01 - 1.20)
Nouvelle-Aquitaine	1.25 (1.14 – 1.36)	1.19 (1.09 - 1.31)	1.26 (1.14 - 1.38)
Normandie	1.17 (1.06 – 1.29)	1.16 (1.05 - 1.29)	1.18 (1.06 - 1.32)
Pays de la Loire	1.21 (1.09 – 1.35)	1.18 (1.06 - 1.32)	1.26 (1.13 - 1.41)
Bretagne	1.18 (1.03 – 1.35)	1.11 (0.97 - 1.28)	1.22 (1.07 - 1.40)
Corse	1.25 (0.96 – 1.64)	1.26 (0.96 - 1.66)	1.32 (1.00 - 1.75)
**Social deprivation index (quintiles)**			
1 : least deprived	1	1	1
2	1.00 (0.94 – 1.06)	1.00 (0.94 - 1.06)	1.00 (0.94 - 1.06)
3	0.98 (0.92 – 1.04)	0.98 (0.93 - 1.05)	0.98 (0.92 - 1.04)
4	0.96 (0.91 – 1.02)	0.97 (0.91 - 1.03)	0.96 (0.90 - 1.02)
5 : most deprived	0.93 (0.88 – 0.99)	0.93 (0.88 - 0.99)	0.92 (0.86 - 0.98)
Unknown	1.00 (0.87 – 1.14)	1.00 (0.88 - 1.15)	1.01 (0.88 - 1.16)
**Addictive disorders**			
Smoking cessation program	1.34 (1.24 – 1.45)	1.33 (1.23 - 1.44)	1.14 (1.05 - 1.25)
Alcohol related disorders	1.63 (1.45 – 1.82)	1.61 (1.43 - 1.80)	1.59 (1.41 - 1.80)
Opioid related disorders	0.95 (0.65 – 1.38)	0.98 (0.67 - 1.43)	0.91 (0.60 - 1.38)
**SARS-CoV-2 vaccination before index date**			
Unvaccinated	1	1	1
1 dose	1.00 (0.92 – 1.09)	0.99 (0.91 - 1.08)	1.04 (0.95 - 1.13)
2 doses	1.04 (0.94 – 1.15)	1.03 (0.93 - 1.14)	1.04 (0.94 - 1.16)

	Multivariable model, adjusted hazard ratio (95% CI)	Multivariable model without in situ cancers, adjusted hazard ratio (95% CI)	Multivariable model without lung cancers, adjusted hazard ratio (95% CI)
3 doses	..	..	..
**Immunosuppressive/corticoid treatment**			
Immunosuppressive medication	1.14 (0.93 – 1.39)	1.14 (0.93 - 1.39)	1.00 (0.80 - 1.24)
Oral corticosteroids medication	1.20 (1.02 – 1.42)	1.19 (1.01 - 1.41)	1.20 (1.01 - 1.43)
**Prior cardiometabolic comorbidities**			
Diabetes	0.94 (0.89 – 0.99)	0.95 (0.90 - 1.00)	0.95 (0.90 - 1.01)
Morbid obesity	0.85 (0.67 – 1.07)	0.85 (0.67 - 1.08)	0.89 (0.70 - 1.14)
Dyslipidemia and lipid-lowering treatments	1.02 (0.97 – 1.07)	1.02 (0.97 - 1.07)	1.00 (0.95 - 1.05)
Inherited metabolic diseases or amyloidosis	0.87 (0.61 – 1.22)	0.89 (0.63 - 1.25)	0.95 (0.67 - 1.34)
Hypertension	1.20 (1.15 – 1.25)	1.20 (1.15 - 1.25)	1.22 (1.17 - 1.28)
Coronary heart disease	0.96 (0.90 – 1.03)	0.96 (0.90 - 1.03)	0.93 (0.87 - 1.00)
Obliterative arteriopathy of the lower limbs	1.48 (1.36 – 1.62)	1.49 (1.36 - 1.63)	1.24 (1.12 - 1.38)
Heart rate and conduction disorders	1.07 (1.00 – 1.14)	1.07 (1.00 - 1.15)	1.09 (1.02 - 1.17)
Heart failure	1.00 (0.90 – 1.11)	1.01 (0.90 - 1.13)	1.00 (0.89 - 1.12)
Cardiac valve diseases	0.99 (0.88 – 1.12)	1.00 (0.89 - 1.13)	1.03 (0.91 - 1.17)
Stroke	1.01 (0.91 – 1.11)	1.01 (0.91 - 1.12)	1.01 (0.91 - 1.12)
Other cardiovascular diseases	1.16 (1.01 – 1.34)	1.16 (1.00 - 1.34)	1.17 (1.00 - 1.36)
**Prior respiratory comorbidities**			
Chronic respiratory diseases (excluding cystic fibrosis)	1.20 (1.13 – 1.27)	1.20 (1.13 - 1.27)	1.08 (1.02 - 1.16)
Cystic fibrosis	..	..	..
Pulmonary embolism	1.14 (0.90 – 1.43)	1.16 (0.92 - 1.47)	1.12 (0.88 - 1.44)
**Prior inflammatory and skin comorbidities**			
Inflammatory bowel disease	0.85 (0.66 – 1.10)	0.86 (0.67 - 1.11)	0.84 (0.64 - 1.11)
Rheumatoid arthritis and RD	0.91 (0.73 – 1.13)	0.87 (0.69 - 1.10)	0.95 (0.75 - 1.20)
Ankylosing spondylitis and RD	0.87 (0.67 – 1.12)	0.88 (0.68 - 1.15)	0.89 (0.68 - 1.18)
Other inflammatory diseases	0.94 (0.74 – 1.20)	0.96 (0.75 - 1.23)	1.03 (0.80 - 1.32)
Psoriasis	1.16 (0.99 – 1.35)	1.17 (1.00 - 1.37)	1.12 (0.95 - 1.33)
**Prior psychiatric and neurodegenerative comorbidities**			
Neurotic / mood disorders, use of antidepressants	0.91 (0.85 – 0.98)	0.91 (0.85 - 0.98)	0.90 (0.83 - 0.97)
Psychotic disorders, use of neuroleptics	0.99 (0.87 – 1.13)	1.00 (0.88 - 1.15)	0.97 (0.84 - 1.11)
Use of anxiolytics	1.03 (0.96 – 1.10)	1.03 (0.96 - 1.10)	1.00 (0.93 - 1.08)
Use of hypnotics	1.11 (1.02 – 1.21)	1.10 (1.01 - 1.20)	1.09 (0.99 - 1.20)
Psychiatric disorders since childhood	0.25 (0.04 – 1.79)	0.26 (0.04 - 1.84)	..
Epilepsy	0.86 (0.67 – 1.10)	0.88 (0.69 - 1.12)	0.83 (0.63 - 1.08)
Multiple sclerosis	0.62 (0.37 – 1.06)	0.64 (0.38 - 1.09)	0.65 (0.38 - 1.13)
Paraplegia	1.31 (0.93 – 1.86)	1.34 (0.95 - 1.89)	1.32 (0.92 - 1.90)
Myopathy or myasthenia	1.41 (0.92 – 2.17)	1.38 (0.88 - 2.14)	1.53 (0.99 - 2.38)
Parkinson disease	0.97 (0.79 – 1.17)	0.96 (0.79 - 1.17)	1.01 (0.83 - 1.23)
Dementia (including AD)	0.88 (0.72 – 1.08)	0.89 (0.73 - 1.09)	0.89 (0.72 - 1.10)
Mental disability	0.93 (0.59 – 1.49)	0.95 (0.60 - 1.52)	0.97 (0.60 - 1.56)

	Multivariable model, adjusted hazard ratio (95% CI)	Multivariable model without in situ cancers, adjusted hazard ratio (95% CI)	Multivariable model without lung cancers, adjusted hazard ratio (95% CI)
Other psychiatric illnesses	1.12 (0.89 – 1.40)	1.13 (0.90 - 1.41)	1.15 (0.91 - 1.46)
Other neurological diseases	1.21 (0.96 – 1.54)	1.22 (0.96 - 1.55)	1.21 (0.94 - 1.55)
**Other comorbidities**			
HIV infection	1.53 (1.22 – 1.91)	1.52 (1.21 - 1.91)	1.56 (1.23 - 1.97)
Liver diseases	1.47 (1.31 – 1.66)	1.48 (1.31 - 1.67)	1.56 (1.37 - 1.77)
Pancreatic diseases	1.23 (0.98 – 1.53)	1.23 (0.98 - 1.53)	1.22 (0.97 - 1.55)
Chronic dialysis	1.57 (1.16 – 2.12)	1.55 (1.15 - 2.11)	1.61 (1.17 - 2.22)
Kidney transplantation	1.18 (0.80 – 1.75)	1.21 (0.82 - 1.79)	1.26 (0.83 - 1.93)
Cardiac transplantation	1.02 (0.14 – 7.38)	1.04 (0.14 - 7.49)	1.34 (0.19 - 9.71)
Liver transplantation	0.99 (0.24 – 4.00)	1.00 (0.25 - 4.08)	0.61 (0.08 - 4.37)
Lung transplantation	0.77 (0.11 – 5.51)	0.79 (0.11 - 5.65)	1.10 (0.15 - 7.89)
Haemophilia / severe haemostasis disorders	1.46 (0.98 – 2.18)	1.43 (0.95 - 2.16)	1.55 (1.03 - 2.33)
Down syndrome	0.72 (0.18 – 2.89)	0.74 (0.18 - 2.97)	0.76 (0.19 - 3.05)
Other long-term condition	1.18 (1.05 – 1.32)	1.19 (1.06 - 1.33)	1.23 (1.10 - 1.38)

Abbreviations: RD = Related Diseases, AD = Alzheimer Disease.

Supplementary Table S4: Stratification according to cancer site, taking into account diagnoses made after hospital discharge. The multivariable model (aHR) was adjusted for all variables presented in Table 1.

	Cancer in absolute number	Median follow-up in days (q1-q3)	Crude incidence /100,000 Pyr	Multivariable model, with cancer diagnosis after hospital discharge adjusted hazard ratio (95% CI)
**Any malignancy**				
Matched control group	10,907	200 (99 - 322)	1,464	1
ICU hospitalized group	795	189 (100 - 287)	1,943	1.17 (1.08 - 1.26)
**Hematological cancer**				
Matched control group	756	203 (106 - 328)	101	1
ICU hospitalized group	101	160 (76 - 262)	247	2.03 (1.62 - 2.53)
**Non-Hodgkin's lymphoma**				
Matched control group	299	212 (111 - 336)	40	1
ICU hospitalized group	39	157 (73 - 263)	95	1.96 (1.37 - 2.80)
**Hodgkin's lymphoma**				
Matched control group	32	194 (122 - 290)	4	1
ICU hospitalized group	2	204 (135 - 273)	5	1.02 (0.23 - 4.46)
**Myeloma**				
Matched control group	142	198 (92 - 305)	19	1
ICU hospitalized group	13	97 (59 - 284)	32	1.21 (0.67 - 2.21)
**Leukemia**				
Matched control group	283	201 (103 - 323)	38	1
ICU hospitalized group	47	169 (87 - 221)	115	2.67 (1.91 - 3.72)
**Female breast cancer**				
Matched control group	861	191 (98 - 319)	116	1
ICU hospitalized group	53	156 (101 - 218)	130	1.08 (0.81 - 1.45)
**Prostate cancer**				
Matched control group	2,180	200 (97 - 323)	293	1
ICU hospitalized group	102	219 (121 - 303)	249	0.86 (0.70 - 1.05)
**Colon cancer**				
Matched control group	666	195 (103 - 335)	89	1
ICU hospitalized group	60	188 (83 - 286)	147	1.38 (1.05 - 1.82)
**Rectal cancer**				
Matched control group	247	219 (117 - 359)	33	1
ICU hospitalized group	12	175 (53 - 418)	29	0.91 (0.50 - 1.64)
**Lung cancer**				
Matched control group	998	198 (105 - 313)	134	1
ICU hospitalized group	104	175 (98 - 271)	254	1.50 (1.22 - 1.86)
**Liver cancer**				
Matched control group	302	183 (88 - 298)	41	1
ICU hospitalized group	24	218 (83 - 311)	59	0.74 (0.48 - 1.15)
**Bladder cancer**				
Matched control group	579	202 (93 - 323)	78	1
ICU hospitalized group	39	150 (69 - 268)	95	1.02 (0.73 - 1.43)
**Renal cancer**				
Matched control group	301	205 (106 - 338)	40	1
ICU hospitalized group	49	178 (103 - 251)	120	2.67 (1.93 - 3.69)
**Uterine cancer**				
Matched control group	101	180 (112 - 294)	14	1

	Cancer in absolute number	Median follow-up in days (q1-q3)	Crude incidence /100,000 Pyr	Multivariable model, with cancer diagnosis after hospital discharge adjusted hazard ratio (95% CI)
ICU hospitalized group	5	181 (40 - 268)	12	0.90 (0.36 - 2.29)
**Malignant melanoma**				
Matched control group	323	199 (108 - 302)	43	1
ICU hospitalized group	11	213 (80 - 302)	27	0.74 (0.40 - 1.36)
**Other malignancy**				
Matched control group	3,569	202 (96 - 322)	479	1
ICU hospitalized group	235	216 (125 - 331)	574	1.05 (0.91 - 1.20)

Supplementary Table S5: Stratification according to cancer site, taking into account death as a competing risk. The multivariable model (aHR) was adjusted for all variables presented in Table 1.

	Cancer in absolute number	Median follow-up in days (q1-q3)	Crude incidence /100,000 Pyr	Multivariable model, with death as competing risk adjusted hazard ratio (95% CI)
**Any malignancy**				
Matched control group	10,944	168 (73 – 270)	1,469	1
ICU hospitalized group	897	200 (99 – 322)	2,192	1.25 (1.16 - 1.34)
**Hematological cancer**				
Matched control group	759	202 (106 - 327)	102	1
ICU hospitalized group	124	107 (50 - 233)	303	2.42 (1.96 - 2.99)
**Non-Hodgkin's lymphoma**				
Matched control group	300	210 (111 - 334)	40	1
ICU hospitalized group	43	111 (55 - 262)	105	2.05 (1.44 - 2.92)
**Hodgkin's lymphoma**				
Matched control group	32	194 (122 - 290)	4	1
ICU hospitalized group	2	204 (135 - 273)	5	0.98 (0.23 - 4.26)
**Myeloma**				
Matched control group	142	198 (92 - 305)	19	1
ICU hospitalized group	22	53 (6 - 118)	54	2.10 (1.26 - 3.50)
**Leukemia**				
Matched control group	285	201 (103 - 323)	38	1
ICU hospitalized group	57	137 (59 - 203)	139	3.15 (2.28 - 4.34)
**Female breast cancer**				
Matched control group	863	192 (98 - 320)	116	1
ICU hospitalized group	55	154 (92 - 218)	134	1.08 (0.82 - 1.44)
**Prostate cancer**				
Matched control group	2,187	200 (97 - 323)	293	1
ICU hospitalized group	104	216 (117 - 299)	254	0.82 (0.67 - 1.01)
**Colon cancer**				
Matched control group	668	195 (103- 335)	90	1
ICU hospitalized group	75	140 (42 - 271)	183	1.64 (1.27 - 2.11)
**Rectal cancer**				
Matched control group	247	219 (117 - 359)	33	1
ICU hospitalized group	12	175 (53 - 418)	29	0.86 (0.47 - 1.56)
**Lung cancer**				
Matched control group	1,000	197 (105 - 313)	134	1
ICU hospitalized group	118	143 (74 - 244)	288	1.61 (1.32 - 1.98)
**Liver cancer**				
Matched control group	303	182 (88 - 298)	41	1
ICU hospitalized group	27	189 (82 - 310)	66	0.78 (0.52 - 1.18)
**Bladder cancer**				
Matched control group	581	202 (94 - 322)	78	1
ICU hospitalized group	42	143 (64 - 238)	103	1.03 (0.75 - 1.42)
**Renal cancer**				
Matched control group	302	204 (106 - 338)	41	1
ICU hospitalized group	58	140 (80 - 245)	142	3.01 (2.20 - 4.11)
**Uterine cancer**				
Matched control group	103	180 (109 - 299)	14	1
ICU hospitalized group	6	111 (20 - 268)	15	0.98 (0.42 - 2.28)

	Cancer in absolute number	Median follow-up in days (q1-q3)	Crude incidence /100,000 Pyr	Multivariable model, with death as competing risk adjusted hazard ratio (95% CI)
**Malignant melanoma**				
Matched control group	326	199 (108 - 302)	44	1
ICU hospitalized group	11	213 (80 - 302)	27	0.70 (0.38 - 1.28)
**Other malignancy**				
Matched control group	3,581	201 (96 - 321)	481	1
ICU hospitalized group	265	202 (96 - 300)	648	1.13 (0.99 - 1.29)

Supplementary Table S6: List of codes used to classify cancers in the SNDS database.

ICD-10 Code	Cancer site
C50	Female breast cancer
C61	Prostate cancer
C34	Lung cancer
C18, C19	Colon cancer
C20	Rectal cancer
C22	Liver cancer
C67	Bladder cancer
C54, C55	Uterine cancer
C43	Malignant melanoma
C64	Renal cancer
C82 - C88	Non-Hodgkin's lymphoma
C81	Hodgkin's lymphoma
C90	Myeloma
C91 - C96	Leukemia
C00-D09	Any active malignancy (including in situ tumors)

ICD-10 Code	Cancer site
C00-C14	Malignant tumors of the lip, oral cavity and pharynx
C15-C26	Malignant tumors of the digestive organs
C30-C39	Malignant tumors of the respiratory and intrathoracic organs
C40-C41	Malignant tumors of the bones and articular cartilage
C43-C44	Malignant tumors of the skin
C45-C49	Malignant tumors of mesothelial tissue and soft tissue
C50	Malignant breast tumor
C51 - C58	Malignant tumors of female genital organs
C60-C63	Malignant tumors of male genital organs
C64-C68	Malignant tumors of the urinary tract
C69-C72	Malignant tumors of the eye, brain and other parts of the CNS
C73-C75	Malignant tumors of the thyroid and other endocrine glands
C76 - C80	Malignancies of ill-defined, secondary and unspecified sites
C81-C96	Malignant tumors of lymphoid, hematopoietic and related tissues
D00-D09	In situ tumors
